# Occurrence of antibiotic‐resistant bacteria on hydroponically grown butterhead lettuce (*Lactuca sativa var. capitata*)

**DOI:** 10.1002/fsn3.2116

**Published:** 2021-01-23

**Authors:** Warangkana Srichamnong, Natcha Kalambaheti, Susan Woskie, Pornpimol Kongtip, Jintana Sirivarasai, Karl R. Matthews

**Affiliations:** ^1^ Institute of Nutrition Mahidol University Phutthamonthon, Nakhon Pathom Thailand; ^2^ Department of Public Health Zuckerberg College of Health Sciences University of Massachusetts Lowell Lowell MA USA; ^3^ Department of Public Health Mahidol University Bangkok Thailand; ^4^ Nutrition Department Ramathibodi Hospital Phayathai, Bangkok Thailand; ^5^ Department of Food Science Rutgers, The State University of New Jersey New Brunswick NJ USA

**Keywords:** antibiotic, antibiotic‐resistant bacteria, food safety, hydroponics, lettuce

## Abstract

Antibiotics used during production of food crops to control plant diseases may result in selection of antibiotic‐resistant bacteria and occurrence of antibiotic residues. The aim of this research was to evaluate the effect of antibiotics used in butterhead lettuce production on persistence of commensal microbiota. Butterhead lettuce were treated with antibiotics (oxytetracycline, gentamicin, and streptomycin) at different concentrations (100, 200, 300, 400 and 500 ppm) starting at 5 weeks’ growth by spraying once daily for 4 weeks and harvesting 7 days after the final spray application. The population of total aerobic bacteria and antibiotic‐resistant bacteria were determined. The results showed antibiotic usage significantly decreased bacterial populations on lettuce. Moreover, increased concentration of antibiotics resulted in significantly greater decrease in bacterial populations. At a concentration of 500 ppm, all antibiotics achieved an approximate 2 log CFU/g decrease in bacterial populations. A stable population (4 log CFU/g) of potentially antibiotic‐resistant commensal microbiota were maintained throughout production. Screening for level of susceptibility indicated that bacteria exhibited greater resistance to oxytetracycline than gentamicin. In conclusion, application of antibiotics failed to eliminate commensal microbiota, demonstrating large populations of antibiotic‐resistant bacteria reside on lettuce grown under conditions used in the present study. This is the first study focused on antibiotic usage on hydroponic systems. Results of this study suggest regulations directed at antibiotic use on food crops must be developed and implemented to control the selection and spread of antibiotic‐resistant bacteria that present a global health concern.

## Graphical abstract

Multi‐antibiotic‐resistant bacteria identified were not foodborne pathogens. A stable population of antibiotic‐resistant bacteria remained on lettuce treated with antibiotics. Antibiotic residues were detected in a limited number of lettuce samples.

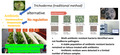

## 1 INTRODUCTION

Agricultural practices in Thailand have shifted dramatically in recent years from traditional to modern monoculture that depends on the use of fertilizers and other agricultural chemicals for increased production. The over‐use of those compounds has had a negative impact on the environment and created health concerns for farmers using those agents (Chaudhary, 2016; Nisha, 2008). In addition to the use of chemical fertilizers and pesticides, farmers are now treating crops with antibiotics to control plant pathogens and increase yields.

The over‐use of antibiotics for the control of plant diseases may result in increased population of antibiotic‐resistant bacteria on crops, which may spread throughout the food chain. The use of antibiotics for control of bacteria on crops may result in antibiotic residues accumulating in the edible portion of crops, agricultural waters, soil, and workers. Large quantities of antibiotics are used annually in livestock production and agriculture operations throughout the world; the eventual fate of antibiotic residues and potential damage to the environmental are now being seriously questioned (Blair et al., 2015; Chaudhary, 2016; Nisha, 2008). The use of antibiotics in production agriculture may adversely impact human health through ingestion of antibiotic residues on edible crops (e.g., lettuce, cabbage). Negative pathological effects include but are not limited to impaired immune response, mutagenicity, reproductive disorders, bone marrow toxicity, and allergy (Nisha, 2008). Ingestion of antibiotic residues may facilitate selection of antibiotic‐resistant bacteria, the emergence, dissemination, and persistence of which represents a major public health concern (Lemus et al., 2008). The indirect impact on human health may include exposure to antibiotic‐resistant bacteria and the altering of normal protective intestinal microbiota. The number and types (genus/species) of antibiotic‐resistant bacteria now detected in patients have increased dramatically. According to the U.S. CDC (2017), antibiotic‐resistant bacteria cause at least 2 million infections, 23,000 deaths per year, and $55–70 billion economic loss per year. In Europe, approximately 25,000 people die annually as the result of multi‐drug‐resistant bacterial infections coupled with a 1.5 billion Euro per year cost to the economy. Antibiotic‐resistant bacteria on food crops include phytopathogens, and spoilage and foodborne bacteria.

In Thailand, farmers regularly use antibiotics to reduce plant disease and increase crop yield (Ministry of public health Thailand, 2005). As a result, many of the crops in Thailand are contaminated with antibiotic residues. Butterhead lettuce (*Lactuca sativa* var. *capitata*) is normally consumed raw in salads. Disease(s) of butterhead lettuce can be caused by viruses including lettuce mosaic virus, fungus such as downy mildew, and bacteria including *Xanthomonas campestris* and *Erwinia carotovora,* which cause black rot or soft rot disease.

Butterhead lettuce in Thailand may be grown indoor, semi‐indoor, or outdoor using hydroponics. Generally, for disease control in hydroponic crops biocontrol agents such as *Trichoderma* are used, but often the causative disease agent is resistant to such treatments (Khalil et al., 2009; Lee & Lee, 2015). The use of antibiotics was viewed as an alternative approach to control plant diseases and replacement of *Trichoderma* treatment. The antibiotics used in this study were gentamicin, oxytetracycline, and streptomycin, which are the antibiotics of choice used by producers in Thailand. According to the regulatory agencies in the USA and Britain, certain antibiotics (streptomycin, oxytetracycline) are permissible for use on production crops including oranges, stone tree fruit, and pome fruit (McManus et al. 2002, British Crop Production Council, 2019). Gentamicin is used in the treatment of animal and human diseases but has been used in crop production to control plant diseases (Vidaver, 2002). This is particularly true in Brazil, Korea, and China for the control of citrus greening disease or huanglongbing and lettuce diseases (Qiao et al., 2018; Vidaver, 2002). Han et al. (2018) reported the highest prevalence of toxigenic *C. difficile* in US retail lettuce. The antibiotic resistance to metronidazole, vancomycin, and erythromycin of the isolated *C. difficile* from retail lettuces could lead to public health concerns.

Antibiotics are substances that inactivate or inhibit bacteria by damaging cell walls, resulting in decreased cytoplasmic synthesis or cell membrane function, or degrading nucleic acids and decreasing protein synthesis (Weledji et al., 2017). Plant diseases linked to phytopathogens can be controlled though the use of antibiotics (British Crop Production Council, 2019). Bacteria may possess intrinsic mechanisms that render resistance to antibiotics or acquire antibiotic‐resistant genes that protect the organism from the action of an antibiotic. This is supported by the increase in antibiotic‐resistant bacteria (WHO, 2013, Ministry of public health, Thailand, 2015, Sultana et al., 2014, Sinegani & Younessi, 2017).

Thailand lacks appropriate regulations to control the use of antibiotics in agricultural crop production; antibiotics are considered a more cost‐effective method than other chemical agents to control plant disease. Unregulated application of antibiotics may result in increased populations of antibiotic‐resistant bacteria (phytopathogens, spoilage and pathogenic bacteria) and antibiotic residues on crops intended for human consumption creating a significant human health risk. The aim of this research was to evaluate the effect of antibiotic use in butterhead lettuce production on antibiotic‐resistant bacterial populations associated with butterhead lettuce and antibiotic residues.

## 2 MATERIALS AND METHODS

### 2.1 Lettuce planting and antibiotic treatment

Butterhead lettuce were grown hydroponically in open space systems in which mesh shading was used. This is typical for hydroponic systems in Thailand. The seedlings were transplanted to hydroponic flats in the spacing of 1.00 × 0.80 m comprising a plot of 25 lettuce heads. Five heads of Butterhead lettuce were treated with one of the predetermined concentrations (100, 200, 300, 400, and 500 ppm) of oxytetracycline (OTC), gentamicin (GEN), or streptomycin (STP) starting at 5 weeks of growth through week eight of growth. The antibiotic concentration selected was based on feedback from growers. Antibiotics were applied using a handheld spray applicator. All control [0 ppm antibiotic] lettuce plants were separated from plants treated with antibiotic. The study was conducted twice over two planting seasons.

Control butterhead plants were treated with *Trichoderma* since during preliminary studies nontreated plants either died or were heavily damaged by infestation. Treating control plants with *Trichoderma* had no impact on the study since the goal of the study was to evaluate antibiotic use on persistence of antibiotic‐resistant bacteria and antibiotic residues on butterhead lettuce. Each row of plants was separated using plastic screens to prevent cross‐contamination. Plants were treated once daily (evening) for 4 weeks and then harvested 7 days later. Application of antibiotic was terminated at the end of week 8, and the lettuce was harvested by hand during week 9. Visual evaluation of the physical appearance of lettuce was performed during harvesting.

### 2.2 Total aerobic bacterial count

Heads of butterhead lettuce were harvested, placed in a cooler, and transported back to the laboratory for processing. Samples were processed by placing 25 g of lettuce into 225 ml of 0.1% peptone water in sterile bags and homogenizing for 3 min using a paddle blender homogenizer. Serial (1:10) dilutions were prepared in 0.1% peptone and 100 µl aliquots spread‐plated for determining total aerobic bacterial count (USDA APHIS, 2005).

### 2.3 Antibiotic susceptibility testing

Bacterial colonies were randomly picked from agar plates and evaluated for antibiotic susceptibility using the disk diffusion assay. Bacteria were incubated in tryptic soy broth (TSB) at 37ºC for 24 hr, and turbidity was adjusted to a 1 McFarland standard. A swab was used to swab the surface of plate count agar (PCA), and then, selected antibiotic impregnated disks were placed on the agar surface. The plates were incubated at 37ºC for 24 hr. Susceptibility to oxytetracycline, streptomycin, and gentamicin was determined. The zones of inhibition were measured and recorded and interpreted as susceptible (*S*), intermediate (*I*), or resistant (*R*) based on Clinical and Laboratory Standards Institute (CLSI) (CLSI, 2016).

### 2.4 Bacteria identification by 16S rDNA sequencing

Isolated bacterial colonies were harvested and prepared for shipping to the Thailand Bioresource Research Center (TBRC) for identification using 16S rDNA sequencing. Direct sequencing of the single‐banded and purified PCR products (ca. 1,500 bases, on 16S rDNA by the *E. coli* numbering system; Brosius et al., 1981) was carried out. Sequencing of the purified PCR products was performed on an ABI Prism® 3730XL DNA Sequencer (Applied Biosystems, Foster City, California, USA) by the sequencing service provider. The two primers 27F (5′‐AGA GTT TGA TCM TGG CTC AG‐3′) or 800R (5′‐TAC CAG GGT ATC TAA TCC‐3′) and 518F (5′‐CCA GCA GCC GCG GTA ATA CG‐3′) or 1492R (5′‐TAC GGY TAC CTT GTT ACG ACT T‐3′) (Brosius et al., 1981) for single strand 16S rDNA sequencing, and 4 primers of 27F, 518F, 800R, and 1492R for double strand 16S rDNA sequencing were used.

### 2.5 Determination of antibiotic residue using HPLC

#### 2.5.1 Sample extraction

One gram (composite sample of edible portion of the plant) of freeze‐dried butterhead lettuce was extracted with 30 ml mixed solution of acidified acetonitrile and acetone (v:v = 1:1) by vortex mixing (60 s each time) and ultrasonication (15 min each), followed by centrifugation in air‐cooled conditions at 12,000 *g* for 15 min. The supernatant was extracted two additional times, combined, and evaporated using a rotary evaporator evaporated at 35°C until dry. A 1 ml volume of methanol was added to dilute the residue and passed through a 0.2‐µm filter into a 2‐ml amber glass vial, and stored at −18^o^C before analysis.

#### 2.5.2 High‐performance liquid chromatography analysis

Antibiotic residues in the extracts were measured by high‐performance liquid chromatography (HPLC) with an ultraviolet detector and quaternary gradient pump. Chromatographic separation was performed on the Thermo Scientific‐C18 column (5 µm, 4.6 × 250 mm). For oxytetracycline, the mobile phase consisted of methanol (A) and 0.01 mol/L aqueous oxalic acid (B) with the following gradient conditions: 0–50 min, 50%–100%B; 50–60 min, and 100%‐50%B. The temperature of the column was held on 25°C, the flow rate of the mobile phase was kept at 1.0 ml/min, and the injection volume of the sample was 20 µl. The detection wavelength was set at 360 nm (Xu et al., 2017). For gentamicin and streptomycin, the mobile phase was prepared by mixing methanol, water, and glacial acetic acid (70:25:5) with the isocratic condition. The temperature of the column was held on 25°C, the flow rate of the mobile phase was kept at 1.0 ml/min, and the injection volume of the sample was 20 µl. The detection wavelength was set at 280 nm (Chuong et al., 2013). The limit of detection (LOD) and relative standard deviation (RSD) were 5% and 0.5 ppm, respectively.

### 2.6 Statistical analysis

Analyses were performed in triplicate. All data are subject to an analysis of variance (ANOVA) test (*p* ≤ .05) using SPSS for Windows Ver.16.0 (SPSS Inc., USA).

## 3 RESULTS

### 3.1 Total aerobic bacterial population

The initial total bacterial populations on butterhead lettuce were approximately 6 log CFU/g (Table 1). Following treatment of lettuce with various concentrations of each antibiotic, populations declined 2.0–2.5 log CFU/g depending on the concentration of applied antibiotic. Most samples were acceptable in terms of total bacterial count based on the Thai Department of Medical Science that permits total bacterial counts of less than 6 log CFU/g (DMSC, 2010). Results showed that the use of antibiotics when applied during production can significantly decrease the number of bacteria associated with butterhead lettuce. Increasing an antibiotic concentration from 100 to 500 ppm significantly decreased the number of bacteria associated with the lettuce. At 500 ppm, oxytetracycline, gentamicin, and streptomycin achieved the greatest log decrease in bacterial population of 1.94, 2.47, and 2.13 log CFU/g, respectively. In two instances, control samples had significantly higher bacterial populations than butterhead lettuce treated with low concentration of antibiotics. Although significant, differences are likely of little practical relevance.

**TABLE 1:** Total aerobic bacterial count

Antibiotic	Bacteria (log CFU/g)					
	Control	100 ppm	200 ppm	300 ppm	400 ppm	500 ppm
OTC	6.12 ± 0.01^A^	6.96 ± 0.04^B,a^	5.94 ± 0.01^C,b^	5.56 ± 0.01^D,b^	5.41 ± 0.01^E,a^	4.18 ± 0.01^F,a^
GEN	6.12 ± 0.01^A^	5.80 ± 0.03^B,c^	4.31 ± 0.00^C,c^	4.01 ± 0.01^D,c^	3.97 ± 0.01^D,c^	3.65 ± 0.05^E,c^
STP	6.12 ± 0.01^A^	6.36 ± 0.04^A,b^	6.26 ± 0.01^B,a^	6.25 ± 0.00^B,a^	5.19 ± 0.01^D,b^	3.99 ± 0.01^E,b^

Note

Abbreviations: GEN, gentamicin; OTC, oxytetracycline; STP, streptomycin.

Different letters (A, B, C, D, E, F) in the same row mean that the results are significantly different at *p* ≤ .05 by Duncan's multiple‐range test. ns means no significant difference at *p* > .05.

Different letters (a,b,c) in the same columns mean that the results are significantly different at *p* ≤ .05 by Duncan's multiple‐range test. ns means no significant difference at *p* > .05.

### 3.2 Bacteria identification

Isolated colonies were selected through plating samples on plate count agar (PCA) containing antibiotics (oxytetracycline, gentamicin, or streptomycin) at a concentration of 100 µg/ml. Bacterial colonies that grew on PCA containing antibiotics were selected and streaked onto PCA for isolation. An isolated colony was then selected and sent to the Thailand Bioresource Research Center (TBRC) for identification using 16S rDNA sequencing. Selected bacterial isolates were then subjected to antibiotic susceptibility testing (Tables 2, 3, 4, 5).

**TABLE 2:** Antibiotic susceptibility of bacteria from butterhead lettuce control samples

Bacteria isolate	Antibiotic	Susceptibility			
		50 (µg/ml)	100 (µg/ml)	150 (µg/ml)	200 (µg/ml)
*Pseudomonas spp*	OTC	R	I	S	S
	GEN	R	R	R	R
	STP	R	R	I	I
*Bacillus spp*	OTC	R	S	S	S
	GEN	R	R	R	R
	STP	R	I	I	I
*Sphingomonas sanguinis*	OTC	I	S	S	S
	GEN	R	R	R	R
	STP	R	R	R	R
*Delftia sp*	OTC	I	S	S	S
	GEN	R	R	R	R
	STP	R	R	R	I
*Cupriavidus pauculus*	OTC	S	S	S	S
	GEN	R	R	R	R
	STP	R	R	R	R
*Acinetobacter baumannii*	OTC	I	I	S	S
	GEN	R	R	R	R
	STP	R	R	R	R
*Sphingomonas sanguinis*	OTC	R	R	R	S
	GEN	R	R	R	R
	STP	R	R	R	R

Abbreviations: GEN, gentamicin; I, intermediate; OTC, oxytetracycline; R, resistant; S, susceptible; STP, streptomycin.

**TABLE 3:** Antibiotic susceptibility of bacteria from butterhead lettuce treated with oxytetracycline

Concentration of antibiotic applied to lettuce	Bacterial isolate	Susceptibility			
		50 (µg/ml)	100 (µg/ml)	150 (µg/ml)	200 (µg/ml)
100 ppm	*Acinetobacter baumannii*	R	R	R	R
	*Bacillus spp*	R	R	R	I
200 ppm	*Pseudomonas spp*	R	R	I	I
	*Bacillus spp*	R	R	R	R
	*Acinetobacter baumannii*	R	R	R	R
300 ppm	*Acinetobacter baumannii*	R	R	R	R
	*Bacillus spp*	R	R	R	R
400 ppm	*Acinetobacter baumannii*	R	S	S	S
	*Bacillus spp*	R	I	S	S
	*Pseudomonas spp*	I	S	S	S
500 ppm	*Acinetobacter baumannii*	R	S	S	S
	*Bacillus spp*	I	S	S	S
	*Pseudomonas spp*	S	S	S	S

Abbreviations: GEN, gentamicin; I, intermediate; OTC, oxytetracycline; R, resistant; S, susceptible; STP, streptomycin.

**TABLE 4:** Antibiotic susceptibility of bacteria from butterhead lettuce treated with gentamicin

Concentration of antibiotic applied to lettuce	Bacterial isolate	Susceptibility			
		50 (µg/mL)	100 (µg/ml)	150 (µg/mL)	200 (µg/mL)
100 ppm	*Delftia sp*	R	R	R	R
	*Cupriavidus pauculus*	R	R	R	R
	*Sphingobacterium changzhouense*	R	R	R	R
200 ppm	*Delftia sp*	R	R	R	R
	*Cupriavidus pauculus*	R	R	R	R
	*Sphingobacterium changzhouense*	R	R	R	R
	*Ralstonia insidiosa*	R	R	R	R
300 ppm	*Delftia sp*	R	R	R	R
	*Cupriavidus pauculus*	R	R	R	R
	*Sphingobacterium changzhouense*	R	R	R	R
400 ppm	*Delftia sp*	R	R	R	R
	*Cupriavidus pauculus*	R	R	R	R
	*Sphingobacterium changzhouense*	R	R	R	R
500 ppm	*Delftia sp*	R	R	I	I
	*Cupriavidus pauculus*	R	R	R	R
	*Sphingobacterium changzhouense*	R	R	R	R
	*Ralstonia insidiosa*	R	R	R	R

Abbreviations: GEN, gentamicin; I, intermediate; OTC, oxytetracycline; R, resistant; S, susceptible; STP, streptomycin.

**TABLE 5:** Antibiotic susceptibility of bacteria from butterhead lettuce treated with streptomycin

Concentration of antibiotic applied to lettuce	Bacterial isolate	Susceptibility			
		50 (µg/mL)	100 (µg/ml)	150 (µg/mL)	200 (µg/mL)
100	*Pseudomonas spp*	R	R	R	R
	*Cellulomonas sp*	R	R	R	R
	*Lysinibacillucs fusiformis*	R	R	R	R
	*Acinetobacter baumannii*	R	R	R	R
200	*Pseudomonas spp*	R	R	R	R
	*Cellulomonas sp*	R	R	R	R
	*Lysinibacillucs fusiformis*	R	R	R	R
300	*Cellulomonas sp*	R	R	R	R
	*Lysinibacillucs fusiformis*	R	R	R	R
	*Acinetobacter baumannii*	R	R	R	R
400	*Pseudomonas spp*	R	R	R	R
	*Cellulomonas sp*	R	R	R	R
	*Lysinibacillucs fusiformis*	R	R	R	R
	*Acinetobacter baumannii*	R	R	R	R
500	*Pseudomonas spp*	R	R	I	I
	*Cellulomonas sp*	R	R	R	R
	*Lysinibacillucs fusiformis*	R	R	R	R
	*Acinetobacter baumannii*	R	R	R	R

Abbreviations: GEN, gentamicin; I, intermediate; OTC, oxytetracycline; R, resistant; S, susceptible; STP, streptomycin.

Plating of isolates recovered from control samples onto PCA containing antibiotic resulted in recovery of bacteria including *Acinetobacter, Bacillus, Cupriavidus, Delftia, Sphingomonas*, and *Pseudomonas*. *Sphingomonas sanguinis* resides on the upper leaf surfaces (Kim et al., 1998) and can cause brown spots on yellow Spanish melon fruits (Buonaurio et al., 2002) and bacterial apical necrosis on mango (Liu et al., 2018).

Isolates recovered from lettuce treated with oxytetracycline included *Acinetobacter baumannii*, *Bacillus*, and *Pseudomonas*. *Acinetobacter baumannii* has been linked to respiratory infection, pneumonia, and urinary tract infections (Tiwari et al., 2015). The bacteria can be also found in untreated water (Dekic et al., 2018). Antibiotic‐resistant bacteria recovered from lettuce treated with gentamicin included *Delftia sp, Cupriavidus pauculus, Sphingobacterium changzhouense, and Ralstonia insidiosa*. *Delftia* sp have been shown to be plant growth‐promoting bacteria (PGPB). PGPB promote plant growth either by helping to provide nutrients to the host plant or by helping to resist infection by phytopathogens (Agafonova et al., 2017; Ubalde et al., 2012). *Cupriavidus pauculus* is an environmental bacterium, which may be found in soil, in water, or on plants (Duggal et al., 2013). *Ralstonia insidiosa* is a waterborne bacterium and an emerging pathogen in hospital settings (Ryan & Adley, 2013).

*Acinetobacter, Cellulomonas sp,* and *Lysinibacillus fusiformis* and *Pseudomonas* were isolated from butterhead lettuce treated with streptomycin. *Cellulomonas sp*. and *Lysinibacillucs fusiformis* may also function in growth promotion of plants (Egamberdiyeva & Höflich, 2002; Rafique et al., 2017; Sahu et al., 2018).

### 3.3 Antibiotic susceptibility

Antibiotic susceptibility was determined for each isolate identified (Tables 2, 3, 4, 5). Most isolates recovered from samples collected from control lettuce plants exhibited multiantibiotic resistance (Table 2). All isolates, Gram‐negative and Gram‐positive, exhibited resistance to gentamicin and streptomycin to at least 50 µg/ml of each antibiotic. Only *Pseudomonas* was resistant to the lowest concentration of oxytetracycline, the other isolates were susceptible or exhibited intermediate susceptibility.

Susceptibility to oxytetracycline of isolates recovered from butterhead lettuce treated with oxytetracycline is shown in Table 3. Results indicate that all isolates recovered from butterhead lettuce treated with oxytetracycline at concentrations of 100–300 ppm were resistant to oxytetracycline up to 100 µg/ml. Isolates were susceptible at 400 and 500 ppm oxytetracycline. Antibiotic susceptibility of isolates recovered from lettuce treated with gentamicin or streptomycin exhibited resistance to 200 µg/ml of each antibiotic except for a single *Delftia* isolate and *Pseudomonas* isolate (Tables 4 and 5).

### 3.4 Antibiotic residue

Antibiotic residue associated with butterhead lettuce treated with antibiotics is shown in Table 6. Two plantings of lettuce were grown: Planting 1 was planted in winter (December–January) while Planting 2 was planted in summer (April–May). Butterhead lettuce from Planting 1 treated with oxytetracycline at concentrations 400–500 ppm had residue at 2.24 and 2.71 ppm, respectively. All remaining samples from Planting 1 and Planting 2 antibiotic residues were not detected.

**TABLE 6:** Antibiotic residue associated with treated butterhead lettuce

Concentration of antibiotic applied to lettuce	Oxytetracycline		Gentamicin		Streptomycin	
	Planting 1	Planting 2	Planting 1	Planting 2	Planting 1	Planting 2
Control	ND	ND	ND	ND	ND	ND
100 ppm	ND	ND	ND	ND	ND	ND
200 ppm	ND	ND	ND	ND	ND	ND
300 ppm	ND	ND	ND	ND	ND	ND
400 ppm	2.24 ± 0.05	ND	ND	ND	ND	ND
500 ppm	2.71 ± 0.02	ND	ND	ND	ND	ND

Abbreviation: ND, not detected.

## 4 DISCUSSION

The practice of applying antibiotics directly to crops intended for human consumption creates risks associated with antibiotic residues, maintenance of antibiotic‐resistant bacteria, and selection of antibiotic‐resistant bacteria. Fresh vegetables that are often consumed raw may harbor high populations of antibiotic‐resistant bacteria, serving as a reservoir of resistance genes that can potentially be transferred to bacteria of animal, human, or environmental origin (Falomir et al., 2013). Many people consume fresh vegetables such as spinach and lettuce without applying a process (e.g., cooking) to inactivate bacteria before consumption; this may facilitate the transfer of antibiotic‐resistant gene(s) in the human gastrointestinal tract creating a threat to human health (Schjorring & krogfelt, 2011). The literature is rich with papers on the prevalence of antibiotic‐resistant bacteria associated with fresh produce, but not the ramifications of direct application of antibiotics to food crops such as leafy greens and persistence of antibiotic‐resistant bacteria (Ghafura et al., 2019; Luo et al., 2017).

In the present study, butterhead lettuce was treated with antibiotics and the presence of antibiotic‐resistant bacteria and antibiotic residues determined on mature lettuce heads typically harvested for human consumption. Antibiotic residues were detected in association with a limited number of lettuce samples. Antibiotic‐resistant bacteria were isolated from control (no antibiotic exposure) and treated (oxytetracycline, gentamicin, or streptomycin) lettuce samples. These results are not surprising since antibiotic‐resistant bacteria have been isolated from irrigation water, soil, and soil amendments (Lupo et al., 2012; Wellington et al., 2013). All isolates identified from control plants were resistant to the highest level (200 µg/ml) of gentamicin tested. The research demonstrates the persistence of antibiotic‐resistant bacteria in covered hydroponic growth environments and on lettuce grown under those conditions. The bacteria identified are not foodborne pathogens but may facilitate the spread of antimicrobial‐resistant genes. The prudent use of antibiotics in agricultural crop production and appropriate regulations and implementation of those regulations are required to mitigate the spread of antibiotic‐resistant bacteria to the production environments, soil, water, and importantly humans through crops that are typically consumed raw.

The antibiotics selected for use in the present study have the same mechanism of action. They work by inhibiting protein synthesis by binding the 30S ribosome subunit. Gentamicin and streptomycin are aminoglycoside antibiotics and effective against Gram‐negative and Gram‐positive bacteria. Ultimately, the antibiotics act by inhibiting the ability of the bacterium to maintain a cell wall. Oxytetracycline is a broad‐spectrum antibiotic active against Gram‐positive and Gram‐negative bacteria (Davies & Davies, 2010; Kapoor et al., 2017). Streptomycin was the first antibiotic used effectively in agricultural production. The use of streptomycin is limited since issues have been raised surrounding toxicity and antibiotic resistance. Unfortunately, this has resulted in the use of various other antibiotics, which exasperates the selection of multi‐antibiotic‐resistant bacteria. Most growers in Thailand use oxytetracycline and gentamicin rather than streptomycin or a biocontrol agent such as *Trichoderma*.

Microorganisms can be intrinsically resistant to certain antibiotics because of inherent structural or functional characteristics or acquire resistance (Blair et al., 2015; Lin et al., 2015). Microorganisms acquire resistance gene(s) from other bacteria by mobile plasmids or transposons through horizontal gene transfer (Chellat et al., 2016). This is the most common means by which plant‐associated bacteria have developed antibiotic resistance (Sundin & Wang, 2018). For example, the major mechanisms *P. aeruginosa* use to counter antibiotic attack can be classified into intrinsic, acquired, and adaptive resistance. The intrinsic resistance of *P. aeruginosa* includes low outer membrane permeability, expression of efflux pumps that expel antibiotics out of the cell, and the production of antibiotic‐inactivating enzymes (Pachori et al., 2019).

### 4.1 Effect of antibiotic application on total aerobic plate count

Lettuce treated with even the highest level (500 ppm) of each antibiotic maintained bacterial populations of approximately 4 log CFU/g (Table 1). Gentamicin had the greatest effect in decreasing microbial populations on butterhead lettuce. These results suggest that natural microflora found on lettuce grown under conditions used in the present study are multi‐antibiotic‐resistant. The microbiota of lettuce in the present study may have come from multiple sources including equipment, water, workers, and the surrounding environment. The hydroponic operations used in the present study are open to the environment with typically only a canopy to mitigate direct exposure of crops to the sun. In Thailand, farmers may use *Trichoderma* as a biocontrol agent to control plant diseases (Harman, 2006). *Trichoderma* acts to control deleterious plant diseases and aids in promoting plant growth and plant defensive mechanisms (Qualhato et al., 2013). Regardless, many farmers believe antibiotics provide better outcomes with respect to minimizing crop loss.

*Bacillus* spp. and *Pseudomonas* spp. are often part of the normal commensal microbiota of plants (Khalil et al., 2009). They are associated with enhanced plant growth and disease prevention through control of phytopathogens (Lee & Lee, 2015). Nonpathogenic *Bacillus* spp. and *Pseudomonas* spp. are used as biocontrol agents in hydroponic operations (Compant et al., 2005). However, their capacity to prevent disease is minimal compared with application of antibiotics.

### 4.2 Bacteria recovered and antibiotic susceptibility

In this study, bacterial isolates recovered were resistant to the antibiotics applied to butterhead lettuce (Li & Webster, 2018). The bacteria ranged from *Acinetobacter* to *Bacillus* to *Pseudomonas* to *Sphingobacterium*, and are considered spoilage or normal commensal microbiota of plants. Not surprisingly, antibiotic‐resistant bacteria were recovered from control plants (Table 2). One strain of *S. sanguinis* was resistant to all concentrations of each antibiotic except 200 µg/ml oxytetracycline.

Results from total aerobic count and identified species of bacteria demonstrate high populations of antibiotic‐resistant bacteria reside on lettuce cultivated under conditions used in the present study. Potentially, approximately 4 log CFU/g bacterial populations on butterhead lettuce are resistant to exposure to 500 ppm oxytetracycline, gentamicin, or streptomycin (Table 1). Individual strains recovered from plants exposed to even the highest level (500 ppm) of an antibiotic typically exhibited resistance to 200 µg/ml of that same antibiotic. The exception was for isolates recovered from lettuce treated with 400 ppm and 500 ppm oxytetracycline.

All selected bacterial isolates identified from control plants exhibited resistance to 200 µg/ml gentamicin (Table 2). All but one of the isolates recovered from plants treated with gentamicin exhibited resistance to the antibiotic. All isolates recovered from lettuce treated with streptomycin were resistant to streptomycin (Table 5), although not all isolates recovered from control plants were resistant to streptomycin. These results suggest that exposure of lettuce commensal bacteria to antibiotics may lead to selection of strains resistant to the applied antibiotic. Although the bacteria recovered in the present study were not foodborne pathogens, they still enter the food chain and may contribute to the spread of antimicrobial resistance.

The spread of antimicrobial‐resistant bacteria through food represents a significant human health concern even when the bacteria are not considered as foodborne pathogens. Commensal microbiota may exchange and spread genes that encode for antimicrobial resistance to spoilage bacteria and foodborne pathogens. The transfer of the extended‐spectrum β‐lactamase‐encoding gene (blaSHV18) among *Klebsiella pneumoniae* recovered from seed sprouts was investigated (Jung & Matthews, 2016). The study demonstrated that transconjugants carried the *bla*_SHV18_ gene and cotransfer of a class 1 integrase gene and resistance to tetracycline, trimethoprim/sulfamethoxazole occurred during mating. Others have demonstrated that streptogramin‐resistant enterococci are present in deli salads (Christensen et al., 2008). Subsequent research demonstrated that native salad vancomycin‐sensitive *Enterococcus* disseminated *vanA* to streptogramin‐resistant enterococci of food and animal origin (Christensen & Matthews, 2011). Conditions (food matrix, temperature) that bacteria may exchange genes encoding for antibiotic resistance likely vary substantially.

### 4.3 Effect of antibiotic application on antibiotic residues

Antibiotic residues on crops may have a direct negative impact on human health through altering of normal gut protective flora (Lemus et al., 2008). According to McManus et al. (2002), the concentration of antibiotics commonly used in crop production is 50–300 ppm. The physical and chemical properties of each antibiotic used in the present study may have influenced lettuce plant uptake. Gentamicin is highly water‐soluble; 100,000 mg gentamicin can be dissolved in a liter of water. Streptomycin is highly water‐soluble (12,800 mg/L) with a low Kd value (Distribution coefficient) of 8–290 L/kg, properties that enhance mobility and solubility in water. While oxytetracycline has lower water solubility (1,000 mg/L) and a higher Kd value (417–1,026 L/kg), its mobility and solubility is less than gentamicin (Cycoń et al., 2019; Kumar et al., 2005). Mode of application (spray, drip line, furrow) of antibiotics to crops may aid in the contamination of agricultural waters. In this study, farmers used sprinklers to treat plants with antibiotics and to reduce temperature and transpiration of lettuce plants.

Antibiotic residues were only detected in two of the samples evaluated in the present study (Table 6). Biodegradation of antibiotics is influenced by temperature (typical winter and summer growing season temperature in Thailand is 32°C) and accelerated by photodegradation (Hu et al., 2010; Kumar et al., 2005). Hu et al. (2010) did not detect antibiotic residues in plants that were grown and harvested during summer months. Results of the present study may be viewed as encouraging with a limited number of plants positive for antibiotic residues. Of concern is whether the applied antibiotics entered agricultural waters and soil associated with the outdoor hydroponic growing environment.

In the present study, a Pearson correlation analysis provided correlations between antibiotic residue and total aerobic bacterial count. Results show no statistically significant correlation between the residue of antibiotics and total aerobic bacterial count. These results were not unexpected since the butterhead lettuce harbored high population of antibiotic‐resistant commensal microbiota.

The use of oxytetracycline, gentamicin, and streptomycin on crops intended for human consumption raises concerns over toxicity and bacterial resistance. In this study, the population of antibiotic‐resistant bacteria remained high (approx. 4 log CFU/g) even on lettuce treated with the highest concentration of each antibiotic evaluated. The prevalence of bacterial resistance to aminoglycoside antibiotics was greater than tetracycline antibiotics. Antibiotic residues on crops remain a concern although only two samples were found positive in the present study. Government regulatory agencies must be proactive in developing strategies aimed at preventing the "off‐label" use of antibiotics in crop production. Natural antimicrobials offer an alluring alternative for control of plant diseases.

## CONFLICT OF INTEREST

The authors have no conflicts of interested to declare.

## Data Availability

Research data are not shared.

## References

[fsn32116-bib-0001] Agafonova, N. V. , Doronina, N. V. , Kaparullina, E. N. , Fedorov, D. N. , Gafarov, A. B. , Sazonova, O. I. , Sokolov, S. L. , & Trotsenko, Y. A. (2017). A novel Delftia plant symbiont capable of autotrophic methylotrophy. Microbiology, 86, 96–105. 10.1134/S0026261717010039 30207147

[fsn32116-bib-0002] Animal and Plant Health Inspection Service U.S. Department of Agriculture (USDA APHIS) (2005). Standard Operating Procedures. Standard Bacterial Plate Count. https://www.aphis.usda.gov/animal_health/vet_biologics/publications/BBSOP0019.pdf

[fsn32116-bib-0003] Blair, J. M. , Webber, M. A. , Baylay, A. J. , Ogbolu, D. O. , & Piddock, L. J. (2015). Molecular mechanisms of antibiotic resistance. Nature Reviews Microbiology, 13, 42–51. 10.1038/nrmicro3380 25435309

[fsn32116-bib-0004] British crop production council (2019). The Pesticide Manual 18th Edition. London British Crop Production Council. 1 January 2018.

[fsn32116-bib-0005] Brosius, J. , Dull, T. J. , Sleeter, D. D. , & Noller, H. F. (1981). Gene organization and primary structure of a ribosomal RNA operon from *Escherichia coli* . Journal of Molecular Biology, 148, 107–127. 10.1016/0022-2836(81)90508-8 7028991

[fsn32116-bib-0006] Buonaurio, R. , Stravato, V. M. , Kosako, Y. , Fujiwara, N. , Naka, T. , Kobayashi, K. , Cappelli, C. , & Yabuuchi, E. (2002). *Sphingomonas melonis* sp. nov., a novel pathogen that causes brown spots on yellow Spanish melon fruits. International Journal of Systematic and Evolutionary Microbiology, 52(6), 2081–2087. 12508872 10.1099/00207713-52-6-2081

[fsn32116-bib-0007] Chaudhary, A. S. (2016). A review of global initiatives to fight antibiotic resistance and recent antibiotics׳ discovery. Acta Pharmaceutica Sinica B, 6, 552–556. 10.1016/j.apsb.2016.06.004 27818921 PMC5071619

[fsn32116-bib-0008] Chellat, M. F. , Raguž, L. , & Riedl, R. (2016). Targeting antibiotic resistance. Angewandte Chemie International Edition, 55, 6600–6626. 10.1002/anie.201506818 27000559 PMC5071768

[fsn32116-bib-0009] Christensen, E. A. , Joho, K. , & Matthews, K. R. (2008). Streptogramin resistance and virulence determinants in vancomycin‐susceptible enterococci isolated from blended deli salads. Journal of Applied Microbiology, 104, 1260–1265. 18248371 10.1111/j.1365-2672.2007.03651.x

[fsn32116-bib-0010] Christensen, E. , & Matthews, K. R. (2011). Secondary transfer and expression of vanA in *Enterococcus faecium* derived from a commensal vancomycin‐susceptible *E. faecium* multi‐component food isolate. Microbial Drug Resistance, 17, 369–376. 21563943 10.1089/mdr.2010.0072

[fsn32116-bib-0011] Compant, S. , Duffy, B. , Nowak, J. , Clément, C. , & Barka, E. A. (2005). Use of Plant Growth-Promoting Bacteria for Biocontrol of Plant Diseases: Principles, Mechanisms of Action, and Future Prospects. Applied and Environmental Microbiology, 71(9), 4951–4959. 10.1128/AEM.71.9.4951-4959.2005 16151072 PMC1214602

[fsn32116-bib-0012] Chuong, M. C. , Chin, J. , Han, J. W. , Kim, E. , Alhomayin, W. , Al Dosary, F. , Rizg, W. , Moukhachen, O. , & Williams, D. A. (2013). High performance liquid chromatography of gentamicin sulfate reference standards and injection USP. International Journal of Pharmaceuticals Analysis, 4, 25–29.

[fsn32116-bib-0013] CLSI (2016). Standard operating policy/procedure standard bacterial plate count. CLSI, Performance Standards for Antimicrobial Susceptibility Testing (CLSI supplement M100S). Clinical and Laboratory Standards Institute, USDA APHIS.

[fsn32116-bib-0014] Cycoń, M. , Mrozik, A. , & Piotrowska‐Seget, Z. Antibiotics in the soil environment—Degradation and their impact on microbial activity and diversity. Frontiers in Microbiology, 338, 1–45. 10.3389/fmicb.2019.00338 PMC6418018 30906284

[fsn32116-bib-0015] Davies, J. , & Davies, D. (2010). Origins and evolution of antibiotic resistance. Microbiology and Molecular Biology Reviews, 74, 417–433. 10.1128/MMBR.00016-10 20805405 PMC2937522

[fsn32116-bib-0016] Dekic, S. , Hrenovic, J. , Ivankovic, T. , & Van Wilpe, E. (2018). Survival of ESKAPE pathogen *Acinetobacter baumannii* in water of different temperatures and pH. Water Science and Technology, 78, 1370–1376. 10.2166/wst.2018.409 30388093

[fsn32116-bib-0017] Department of medical science (DMSC) (2010). The manual for microbial standard in foods. http://food.fda.moph.go.th/data/tradermain/Manual_Of_Law364(Update30‐6‐16).pdf

[fsn32116-bib-0018] Duggal, S. , Gur, R. , Nayar, R. , Rongpharpi, S. R. , Jain, D. , & Gupta, R. K. (2013). Cupriavidus pauculus (*Ralstonia paucula*) concomitant meningitis and septicemia in a neonate: First case report from India. Indian Journal of Medical Microbiology, 31, 405–409. 10.4103/0255-0857.118871 24064653

[fsn32116-bib-0019] Egamberdiyeva, D. , & Höflich, G. (2002). Root colonization and growth promotion of winter heat and pea by Cellulomonas s at different temperatures. Plant Growth Regulation, 38, 219–224.

[fsn32116-bib-0020] Falomir, M. P. , Rico, H. , & Gozalbo, D. (2013). *Enterobacter* and *Klebsiella* species isolated from fresh vegetables marketed in Valencia (Spain) and their clinically relevant resistances to chemotherapeutic agents. Foodborne Pathogens and Disease, 10, 1002–1007. 23980710 10.1089/fpd.2013.1552

[fsn32116-bib-0021] Ghafura, A. , Shankarb, C. , GnanaSoundaria, P. , Venkatesanb, M. , Manib, D. , Thirunarayanana, M. A. , & Veeraraghavan, B. (2019). Detection of chromosomal and plasmid‐mediated mechanisms of colistin resistance in *Escherichia coli* and *Klebsiella pneumoniae* from Indian food samples. Journal of Global Antimicrobial Resistance, 16, 48–52. 10.1016/j.jgar.2018.09.005 30244040

[fsn32116-bib-0022] Han, Y. , King, J. , & Janes, M. E. (2018) Detection of antibiotic resistance toxigenic *Clostridium difficile* in processed retail lettuce. Food Quality and Safety, 2(1), 37–41.

[fsn32116-bib-0023] Harman, G. E. (2006). Overview of mechanisms and uses of Trichodermas. Phytopathology, 96, 190–194. 18943924 10.1094/PHYTO-96-0190

[fsn32116-bib-0024] Hu, X. , Zhou, Q. , & Luo, Y. (2010). Occurrence and source analysis of typical veterinary antibiotics in manure, soil, vegetables and groundwater from organic vegetable bases, northern China. Environmental Pollution, 2992–2998. 10.1016/j.envpol.2010.05.023 20580472

[fsn32116-bib-0025] Jung, Y. , & Matthews, K. R. (2016). Transfer of extended spectrum β‐lactamase encoding gene, bla_shv18_ gene, between *Klebsiella pneumoniae* in temperature abused raw foods. Food Microbiology., 60, 39–48. 27554144 10.1016/j.fm.2016.06.002

[fsn32116-bib-0026] Kapoor, G. , Saigal, S. , & Elongavan, A. (2017). Action and resistance mechanisms of antibiotics: A guide for clinicians. Journal of Anaesthesiology, Clinical Pharmacology, 33, 300. 10.4103/joacp.JOACP_349_15 PMC5672523 29109626

[fsn32116-bib-0027] Khalil, S. , Hultberg, M. , & Alsanius, B. W. (2009). Effects of growing medium on the interactions between biocontrol agents and tomato root pathogens in a closed hydroponic system. The Journal of Horticultural Science and Biotechnology, 84, 489–494. 10.1080/14620316.2009.11512553

[fsn32116-bib-0028] Kim, H. , Nishiyama, M. , Kunito, T. , Senoo, K. , Kawahara, K. , Murakami, K. , & Oyaizu, H. (1998). High population of Sphingomonas species on plant surface. Journal of Applied Microbiology, 85, 731–736.

[fsn32116-bib-0029] Kumar, K. , Gupta, S. C. , Chander, Y. , & Singh, A. K. (2005). Antibiotic use in agriculture and its impact on the terrestrial environment. Advances in Agronomy, 87, 1–54.

[fsn32116-bib-0030] Lee, S. , & Lee, J. (2015). Beneficial bacteria and fungi in hydroponic systems: Types and characteristics of hydroponic food production methods. Scientia Horticulturae, 2015(195), 206–215. 10.1016/j.scienta.2015.09.011

[fsn32116-bib-0031] Lemus, J. Á. , Blanco, G. , Grande, J. , Arroyo, B. , García‐Montijano, M. , & Martínez, F. (2008). Antibiotics threaten wildlife: Circulating quinolone residues and disease in avian scavengers. PLoS One, 3, e1444. 10.1371/journal.pone.0001444 18197254 PMC2186382

[fsn32116-bib-0032] Li, B. , & Webster, T. J. (2018). Bacteria antibiotic resistance: New challenges and opportunities for implant‐associated orthopedic infections. Journal of Orthopaedic Research, 36, 22–32. 10.1002/jor.23656 28722231 PMC5775060

[fsn32116-bib-0033] Lin, J. , Nishino, K. , Roberts, M. C. , Tolmasky, M. , Aminov, R. I. , & Zhang, L. (2015). Mechanisms of antibiotic resistance. Frontiers in Microbiology, 6, 34. 10.3389/fmicb.2015.00034 25699027 PMC4318422

[fsn32116-bib-0034] Liu, F. , Zhan, R.‐L. , & He, Z.‐Q. (2018). first report of bacterial dry rot of mango caused by *Sphingomonas sanguinis* in China. Plant Disease, 102, 2632.

[fsn32116-bib-0035] Luo, J. , Yao, X. , Lv, L. , Doi, Y. , Huang, X. , Huang, S. , & Liua, J. H. (2017). Emergence of *mcr‐1* in *Raoultella ornithinolytica* and *Escherichia coli* isolates from retail vegetables in China. Antimicrobial Agents and Chemotherapy, 61(10), e01139–e1217. 10.1128/AAC.01139-17 28739785 PMC5610531

[fsn32116-bib-0036] Lupo, A. , Coyne, S. , & Berendonk, T. U. (2012). Origin and evolution of antibiotic resistance: The common mechanisms of emergence and spread in water bodies. Frontiers in Microbiology, 3, 18. 10.3389/fmicb.2012.00018 22303296 PMC3266646

[fsn32116-bib-0037] McManus, P. S. , Stockwell, V. O. , Sundin, G. W. , & Jones, A. L. (2002). Antibiotic use in plant agriculture. Annual Review of Phytopathology, 40, 443–465. 10.1146/annurev.phyto.40.120301.093927 12147767

[fsn32116-bib-0038] Nisha, A. R. (2008). Antibiotic residues‐a global health hazard. Veterinary World, 1, 375. 10.5455/vetworld.2008.375-377

[fsn32116-bib-0039] Pachori, P. , Gothalwal, R. , & Gandhi, P. (2019). Emergence of antibiotic resistance *Pseudomonas aeruginosa* in intensive care unit; a critical review. Genes Discovery, 6, 109–119. 10.1016/j.gendis.2019.04.001 PMC6545445 31194018

[fsn32116-bib-0041] Qiao, M. , Ying, G. G. , Singerd, A. C. , & Zhu, Y. G. (2018). Review of antibiotic resistant. Environment International, 110, 160–172. 29107352 10.1016/j.envint.2017.10.016

[fsn32116-bib-0042] Qualhato, T. F. , Lopes, F. A. C. , Steindorff, A. S. , Brandao, R. S. , Jesuino, R. S. A. , & Ulhoa, C. J. (2013). Mycoparasitism studies of Trichoderma species against three phytopathogenic fungi: Evaluation of antagonism and hydrolytic enzyme production. Biotechnology Letters, 35, 1461–1468. 23690037 10.1007/s10529-013-1225-3

[fsn32116-bib-0043] Rafique, M. , Sultan, T. , Ortas, I. , & Chaudhary, H. J. (2017). Enhancement of maize plant growth with inoculation of phosphate‐solubilizing bacteria and biochar amendment in soil. Soil Science and Plant Nutrition, 63, 460–469. 10.1080/00380768.2017.1373599

[fsn32116-bib-0044] Ryan, M. P. , & Adley, C. C. (2013). The antibiotic susceptibility of water‐based bacteria *Ralstonia pickettii* and *Ralstonia insidiosa* . Journal of Medical Microbiology, 62, 1025–1031. 10.1099/jmm.0.054759-0 23579396

[fsn32116-bib-0045] Sahu, P. K. , Shivaprakash, M. K. , Mallesha, B. C. , Subbarayappa, C. T. , & Brahmaprakash, G. P. (2018). Effect of bacterial *Endophytes Lysinibacillus* sp. on plant growth and fruit yield of tomato (*Solanum lycopersicum*). International Journal of Current Microbiology and Applied Sciences, 7(05), 3399–3408.

[fsn32116-bib-0046] Schjorring, S. , & Krogfelt, K. A. (2011). Assessment of bacterial antibiotic resistance transfer in the gut. International Journal of Microbiology, 2011, 1–10. 10.1155/2011/312956 PMC3034945 21318188

[fsn32116-bib-0047] Sinegani, A. A. S. , & Younessi, N. (2017). Antibiotic resistance of bacteria isolated from heavy metal‐polluted soils with different land uses. Journal of Global Antimicrobial Resistance, 10, 247–255. 10.1016/j.jgar.2017.05.012 28732786

[fsn32116-bib-0048] Sultana, F. , Afroz, H. , Jahan, A. , Fakruddin, M. D. , & Datta, S. (2014). Multi–antibiotic resistant bacteria in frozen food (ready to cook food) of animal origin sold in Dhaka, Bangladesh. Asian Pacific Journal of Tropical Biomedicine, 4, S268–S271. 10.12980/APJTB.4.2014B85 25183094 PMC4025334

[fsn32116-bib-0049] Sundin, G. W. , & Wang, N. (2018). Antibiotic resistance in plant‐pathogenic bacteria. Annual Review of Phytopathology, 56, 161–180. 10.1146/annurev-phyto-080417-045946 29856934

[fsn32116-bib-0050] Tiwari, V. , Roy, R. , & Tiwari, M. (2015). Antimicrobial active herbal compounds against *Acinetobacter baumannii* and other pathogens. Frontiers in Microbiology, 6, 618. 26150810 10.3389/fmicb.2015.00618 PMC4471432

[fsn32116-bib-0051] Ubalde, M. C. , Braña, V. , Sueiro, F. , Morel, M. A. , Martínez‐Rosales, C. , Marquez, C. , & Castro‐Sowinski, S. (2012). The versatility of Delftia sp. isolates as tools for bioremediation and biofertilization technologies. Current Microbiology, 64, 597–603. 10.1007/s00284-012-0108-5 22476956

[fsn32116-bib-0052] Vidaver, A. K. (2002). Uses of antimicrobials in plant agriculture. Clinical Infectious Diseases, 34, S107–S110. 10.1086/340247 11988880

[fsn32116-bib-0053] Weledji, E. P. , Weledji, E. K. , Assob, J. C. , & Nsagha, D. S. (2017). Pros, cons and future of antibiotics. New Horizons in Translational Medicine, 4, 9–14. 10.1016/j.nhtm.2017.08.001

[fsn32116-bib-0054] Wellington, E. M. , Boxall, A. B. , Cross, P. , Feil, E. J. , Gaze, W. H. , & Hawkey, P. M. (2013). The role of the natural environment in the emergence of antibiotic resistance in Gram‐negative bacteria. The Lancet Infectious Diseases, 13, 155–165. 23347633 10.1016/S1473-3099(12)70317-1

[fsn32116-bib-0055] World Health Organization (WHO) (2013). Antibiotic resistance. https://www.who.int/news‐room/fact‐sheets/detail/antibiotic‐resistance.

[fsn32116-bib-0056] Xu, H. , Mi, H. Y. , Guan, M. M. , Shan, H. Y. , Fei, Q. , Huan, Y. F. , Zhang, Z. Q. , & Feng, G. D. (2017). Residue analysis of tetracyclines in milk by HPLC coupled with hollow fiber membranes‐based dynamic liquid‐liquid micro‐extraction. Food Chemistry, 232, 198. 28490065 10.1016/j.foodchem.2017.04.021

